# Draft genome sequences of six *Rothia mucilaginosa* strains assembled from the human oral microbiome

**DOI:** 10.1128/mra.00508-25

**Published:** 2025-09-30

**Authors:** Daniel Saito, Cristiane Pereira Borges Saito, Fabiana De Souza Cannavan, Siu Mui Tsai

**Affiliations:** 1 Computational Biology Laboratory, Superior School of Health Sciences, Amazonas State University 468981, Manaus, Brazil; 2 Functional Genomics Laboratory, Superior School of Health Sciences, Amazonas State University 468981, Manaus, Brazil; 3 Cellular and Molecular Biology Laboratory, Center for Nuclear Energy in Agriculture, University of Sao Paulo 28133, São Paulo, Brazil; Loyola University Chicago, Chicago, llinois, USA

**Keywords:** *Rothia mucilaginosa*, oral microbiome, human microbiome, saliva

## Abstract

We report draft metagenome-assembled genomes (MAGs) of six *Rothia mucilaginosa* strains recovered from the oral microbiome of distinct human subjects. MAGs were retrieved according to a species-specific genome mapping approach, displaying high average nucleotide identities (≥95.85%) to *R. mucilaginosa* ATCC 25296’s genome and minimal contamination levels (≤3.75%).

## ANNOUNCEMENT

The oral microbiome harbors a diverse bacterial community, with many members still warranting characterization at the strain level (1). *Rothia* are catalase-negative aerobic gram-positive bacilli of the Micrococcaceae family and commensals of the oral cavity and upper respiratory tract of humans (2). More specifically, *Rothia mucilaginosa* has been linked to oral conditions (3, 4) and may act as an opportunistic pathogen in systemic infections, including bacteremia (5), infective endocarditis (6, 7), peritonitis (8), septic arthritis (9), periprosthetic infections (10), and pneumonia (5). Furthermore, the incidence of *R. mucilaginosa*-associated complications appears to be rising, possibly as a reflection of the increased adoption of medical implants, although comprehensive epidemiological data remains lacking (6, 7). Here, we aimed at recovering and characterizing *R. mucilaginosa* metagenome-assembled genomes (MAGs) from human saliva based on a species-directed mapping approach, in an effort to help unveil latent genetic traits critical to disease establishment and progression.

This study was approved by the Ethics Committee of the Amazonas State University (CAAE19521113.0.0000.5016). Twenty-seven volunteers were attended to at the Dental Clinic of the Amazonas State University (Brazil) with no distinction to gender, age, or ethnic background. All participants signed an informed consent adhering to the Declaration of Helsinki (2013). 1.0 mL non-stimulated saliva samples were collected with the OM-505 kit (DNA Genotek), submitted to nucleic acid extraction with the MasterPure Complete DNA and RNA Purification kit (Epicentre) coupled with lysozyme and proteinase K (Thermo Fisher, USA), and fragmented by hydrodynamic sonication. DNA fragments of approximately 300 bp were selected for sequencing on the Illumina HiSeq 4000 platform. To this end, 1.0 µg DNA was used for preparation of subject-specific libraries with the NEBNext Ultra DNA Library Prep Kit according to manufacturer’s instructions, yielding 150 bp paired-end reads. Read quality control was achieved with Novogene’s sequence trimming pipeline, ensuring Q30 ≥85%, and all downstream *in silico* analyses were performed with default software parameters. Reads were merged and adapter sequences excised with PEAR v.0.9.8, while host-related sequences were removed with Bowtie2 (11) by mapping to the GRCh38.p14 human data set (https://www.ncbi.nlm.nih.gov/datasets/genome/GCF_000001405.40). Species-level read mapping to National Center for Biotechnology Information (NCBI)’s genome of *R. mucilaginosa* ATCC 25296 was performed with Bowtie2 (11). Contig assembly and MAGs binning were performed with SPADES v.3.15.5 (12) and Maxbin2. Completeness (50% minimum) and contamination (10% maximum) values were assessed with CheckM v.1.10.18 (13). Taxonomic placement of MAGs was achieved with GTDB-Tk v2.3.2 (14) within Kbase (15) adopting average nucleotide identity (ANI) scores of 95% and 97% for species and strain-level demarcation (16). Genome annotation was conducted with NCBI’s PGAP v.6.7, CARD 4.0.0 (17), and dbCAN3 (18).

A total of 43 MAGs were assembled from 4.79 × 10^8^ raw reads. Among these, six genomes (retrieved from healthy individuals) met the quality standards and were chosen for taxonomic inference and gene annotation. They all displayed ANI values ≥ 95.85% to the reference genome and ≤96.54% among each other, representing distinct *R. mucilaginosa* strains. Overall, carbohydrate-binding modules, carbohydrate esterases, and glycosyl hydrolases/transferases were annotated. CRISPR arrays and DNA integrases/transposases were also unveiled, while Tn elements were absent. Antimicrobial drug resistance determinants were primarily found for macrolides, tetracyclines, fluoroquinolones, penicillins, cephalosporins, phenicols, peptides, rifamycins, lincosamides, glycopeptides, and carbapenems. General annotation features are presented in Table 1.

**TABLE 1:** General information and annotation results of six *R. mucilaginosa* MAGs recovered from non-stimulated saliva of humans

Characteristic	Data for MAG				
	OHS0002	OHS0003	OHS0004	OHS0005	OHS0010
Species-level taxonomy	*R. mucilaginosa*	*R. mucilaginosa*	*R. mucilaginosa*	*R. mucilaginosa*	*R. mucilaginosa*
‍FastANI reference (%)	96.92	96.76	96.04	95.85	96.37
Host clinical condition	Oral health	Oral health	Oral health	Oral health	Oral health
SRA accession no.	SRR14122749	SRR14122738	SRR14122730	SRR14122729	SRR14122724
Biosample accession no.	SAMN44699127	SAMN44699128	SAMN44699129	SAMN44699130	SAMN44699131
Genbank accession no.	JBMDTY000000000.1	JBMDTZ000000000.1	JBMDUA000000000.1	JBMDUB000000000.1	JBMDUC000000000.1
‍Number of reads	4,153,480	3,007,863	5,049,354	3,952,422	5,745,540
‍Number of contigs	294	265	487	261	345
N50 (bp)	7,590	6,171	2,996	15,238	9,508
Genome size (bp)	1,148,813	1,171,221	1,294,581	2,093,715	1,923,565
Genome completeness	53.45%	60.34%	50.00%	98.12%	90.62%
Genome contamination	0%	0%	0%	1.99%	0%
Genome assembly coverage	33.81	29.07	23.79	103.78	203.07
GC content (%)	61.48%	61.47%	61.80%	60.09%	60.04%
Protein encoding genes	1,056	958	1,199	1,725	1,642
RNA genes	21	22	22	58	54
tRNA genes	20	19	21	49	45
ncRNA genes	1	3	1	3	3
Pseudogenes	4	7	3	3	3
CRISPR arrays	0	0	0	1	1
Antibiotic resistance genes	79	65	72	129	127
Carbohydrate-active enzymes	48	38	47	86	81

	OHS0012				
Species-level taxonomy	*R. mucilaginosa*				
‍FastANI reference (%)	96.85				
Host clinical condition	Oral health				
SRA accession no.	SRR14122747				
Biosample accession no.	SAMN44699132				
GenBank accession no.	JBMDUD000000000.1				
‍‍Number of reads	2,116,385				
‍Number of contigs	754				
N50 (bp)	2,559				
Genome size (bp)	1,730,033				
Genome completeness	83.17%				
Genome contamination	3.75%				
Genome assembly coverage	11.36				
GC content (%)	60.14%				
Protein encoding genes	1,713				
RNA genes	36				
tRNA genes	32				
ncRNA genes	2				
Pseudogenes	10				
CRISPR arrays	0				
Antibiotic resistance genes	120				
Carbohydrate-active enzymes	68				

## Data Availability

Reads and biosamples belong to NCBI’s bioproject PRJNA717815 under accession numbers displayed in Table 1.
